# *Fortunella margarita *Transcriptional Reprogramming Triggered by *Xanthomonas citri *subsp. *citri*

**DOI:** 10.1186/1471-2229-11-159

**Published:** 2011-11-11

**Authors:** Abeer A Khalaf, Frederick G Gmitter, Ana Conesa, Joaquin Dopazo, Gloria A Moore

**Affiliations:** 1 Plant Molecular and Cellular Biology Program (PMCB), Horticultural Sciences Department, University of Florida, Gainesville, Fl., 32611,USA; 2 PMCB, Citrus Research and Education Center, University of Florida, Lake Alfred, Fl., USA; 3 Centro de Investigación Príncipe Felipe,Valencia, SPAIN

## Abstract

**Background:**

Citrus canker disease caused by the bacterial pathogen *Xanthomonas citri *subsp. *citri (*Xcc) *has *become endemic in areas where high temperature, rain, humidity, and windy conditions provide a favourable environment for the dissemination of the bacterium. Xcc is pathogenic on many commercial citrus varieties but appears to elicit an incompatible reaction on the citrus relative *Fortunella margarita *Swing (kumquat), in the form of a very distinct delayed necrotic response. We have developed subtractive libraries enriched in sequences expressed in kumquat leaves during both early and late stages of the disease. The isolated differentially expressed transcripts were subsequently sequenced. Our results demonstrate how the use of microarray expression profiling can help assign roles to previously uncharacterized genes and elucidate plant pathogenesis-response related mechanisms. This can be considered to be a case study in a citrus relative where high throughput technologies were utilized to understand defence mechanisms in *Fortunella *and citrus at the molecular level.

**Results:**

**cDNAs from sequenced kumquat libraries (ESTs) made from subtracted RNA populations, healthy vs. infected, were used to make this microarray**. Of 2054 selected genes on a customized array, 317 were differentially expressed (P < 0.05) in Xcc challenged kumquat plants compared to mock-inoculated ones. This study identified components of the incompatible interaction such as reactive oxygen species (ROS) and programmed cell death (PCD). Common defence mechanisms and a number of resistance genes were also identified. In addition, there were a considerable number of differentially regulated genes that had no homologues in the databases. This could be an indication of either a specialized set of genes employed by kumquat in response to canker disease or new defence mechanisms in citrus.

**Conclusion:**

Functional categorization of kumquat Xcc-responsive genes revealed an enhanced defence-related metabolism as well as a number of resistant response-specific genes in the kumquat transcriptome in response to Xcc inoculation. Gene expression profile(s) were analyzed to assemble a comprehensive and inclusive image of the molecular interaction in the kumquat/Xcc system. This was done in order to elucidate molecular mechanisms associated with the development of the hypersensitive response phenotype in kumquat leaves. These data will be used to perform comparisons among citrus species to evaluate means to enhance the host immune responses against bacterial diseases.

## Background

Citrus trees are susceptible to a number of diseases with different degrees of economic impact. One of the most severe in terms of economic losses is citrus canker disease (sometimes referred to as Asiatic citrus canker) caused by *Xanthomonas citri *subsp. *citri*, (synonym, *Xanthomonas axonopodis *pv. *citri *strain A; Xac-A). Xcc is a biotrophic bacterial phytopathogen that belongs to the genus *Xanthomonas *of the α-subdivision v within Proteobacteria. Susceptibility to citrus canker disease varies among citrus types and relatives, but most of the commercially grown citrus types are susceptible hosts to Xcc [1]. Disease symptoms include canker lesions on the green aerial parts of the plant as well as fruit; infections can result in both foliar and fruit abscission, thereby decreasing the productivity of affected trees. In addition there can be reduced profitability as a result of blemished fruit that can be harvested but not sold in the fresh market.

Plants have evolved multiple defence mechanisms to survive pathogen attacks [2]. The first branch of the indispensable plant innate immunity system is triggered by pathogen-associated molecular patterns (PAMPs) such as the lipopolysaccharides (LPSs), peptidoglycan and bacterial flagellin, as well as the chitin and glucan from fungi. The second branch utilizes the nucleotide-binding site-leucine-rich repeat (NBS-LRR) encoded by R (resistance) genes named the effector-triggered immunity (ETI) [3]. The *Xanthomonas *spp. phytobacterial pathogens have evolved unique pathogenesis mechanisms to avoid host recognition and suppress host defences [4,5]. Bacterial effector proteins are delivered via the bacterial type III secretion system (TTSS) into the plant cell to evade recognition by the different plant surveillance systems [6]. These effectors in general contribute to host resistance or susceptibility as well as to modifying host responses. A fundamental element of the ETI in resistant plants is a localized cell collapse or a hypersensitive response (HR) at infection sites in an attempt to restrict the growth of the pathogen [7,8]. This is a common feature of disease resistant responses in incompatible plant-pathogen, and occasionally some non-host, interactions [9,10]. Some of the *Xanthomonas *spp. effector proteins, for instance PthA/AvrBs3, are essential to elicit citrus canker symptoms and if expressed by itself inside host cells, *pthA *is sufficient to cause symptoms of citrus canker disease [11-15]. In the meantime however, other recent studies show that other types of proteins are injected through the Xcc TTSS and do not necessarily alter the physiological and transcriptional responses to the pathogen in citrus [8,10,16,17].

While certain genes involved in systemic acquired resistance (SAR) have been characterized and used as markers for studying plant defence mechanisms [18], crosstalk between signals and hormone pathways has also been proposed [19-21]. Consequently, plant resistance is correlated with the activation of a complex network of defence pathways and the response of the host plant to a microbial assault is therefore expected to result in drastic changes in the patterns of gene expression throughout the plant [22,23].

Kumquats (*Fortunella *spp.), close relatives to citrus species, are reported to have high levels of field resistance to citrus canker [1]. Previously, we have shown a sharply contrasting phenotype in grapefruit and kumquat when both plants were challenged with a high concentration of Xccr (OD600 nm = 0.3) [24]. Grapefruit (*Citrus paradisi *Macf. cv. Duncan), considered to be highly susceptible to the bacterium, showed the characteristic sequence of canker lesion development. Initially lesions appeared as water soaking, followed by the development of a raised corky form; each such lesion is a reservoir of new bacterial inoculum. Bacterial exudates were visible between 10 and 21 days post-inoculation. In contrast, PCD was observed in kumquat leaves in the form of a HR 3-5 days after inoculation with the canker-causing bacterium. Only necrotic lesions were observed and the bacterial population over time was shown to have an 'avirulent' incompatible growth pattern where bacterial multiplication ceased upon the development of necrosis [8,25].

New tools have been developed in recent years through advances in genomics, proteomics, and bioinformatics that have particular utility for examining pathogen: host interaction complexities [22,26-28]. The purpose of this study was to examine simultaneous changes in expression profiles for genes differentially expressed in the early stages (6-72 hpi) of citrus canker infection in kumquat, particularly those previously implicated in PCD-related responses such as HR.

## Results and Discussion

In this study, identification of differentially expressed kumquat genes during its interaction with Xcc was pursued in an attempt to unravel the nature of the resistance mechanism(s) employed by the plant. Previously, kumquat suppression subtractive hybridization (SSH) cDNA libraries were constructed from Xcc-inoculated vs. mock inoculated leaves [24]. Since SSH allows differential amplification of rare target sequences due to the elimination of more abundant house-keeping cDNA transcripts found in common from both samples, the technique has the potential of uncovering pertinent cDNA sequences. Subtraction was done in both directions, forward (inoculated-mock) and reverse (mock-inoculated) and the resulting cDNAs were subsequently sequenced. Preliminary screening macroarrays were used to confirm enrichment of the subtracted libraries with differentially expressed genes (data not shown).

### Microarray experimental design

**Figure 1:**
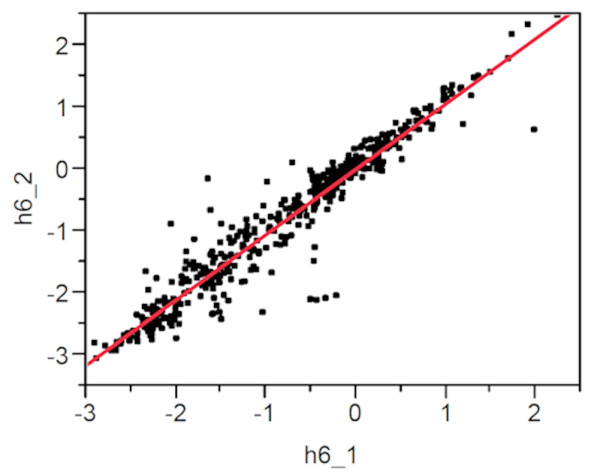
**Scatter plot analysis of the M-values from two microarray hybridizations using RNA samples from two independent kumquat plants inoculated with 5 × 10^8 ^cfu/ml Xcc**. Each spot represents the normalized hybridization signal intensity for each transcript on the microarray. RNA samples from non-inoculated and inoculated leaf tissue were labeled with Cy3 and Cy5, respectively. **(h6_1)**: 6 hours post inoculation hybridization results from slide # 1 hybridized to plant A-RNA samples vs. **(h6_2)**: 6 hours post inoculation hybridization results from slide # 2 hybridized to plant B-RNA samples.

Kumquat microarray chip hybridization data were assessed for overall signal intensity and consistency of the expression ratio over all time points, which resulted in the exclusion of chips with inconsistent results. Figure 1 is a scatter plot showing M-values from two different biological replicate-hybridizations with Xcc-inducible targets (Cy5-labeled) and mock inoculated non-infected targets (Cy3-labeled) confirming high data consistency levels (R^2 ^= 0.921).

### Functional annotation and an overview of global gene expression

The B2GO program [29] was used to assign GO (Gene Ontology) terms for hits obtained through eBLAST homology searches in NCBI. A general view of the similarity of the query set with the NCBI database, the distribution of the cut off for the e-value as well as the distribution of species with similar sequences are shown in Additional files 1, 2, and 3. The GO annotation score is considered to be more intuitive than regular blast e-values since GO annotation is carried out by applying an annotation rule (AR) on the ontology terms. Additionally, query sequence descriptions are obtained by applying a language processing algorithm that extracts informative names and avoids low-content terms such as "hypothetical protein" or "expressed protein". Using Blast2Go suite default parameters, 1042 probes were provided with GO annotations (Additional file 4). Approximately 25% of the transcripts on the array do not show similarity to proteins present in public databases. Some of these could represent exclusive genes of the citrus or kumquat lineages, but a fraction of these uncharacterized sequences may possibly represent low quality or 3'UTR sequences. Similar percentages of unknown sequences have been reported in other small-scale EST projects [30-32] and therefore this pattern can be considered characteristic of this approach. Since a citrus genome sequence is now available, future studies will have a wealth of citrus genomic sequence information that can be utilized to identify kumquat-specific as well as novel citrus genes involved in diverse defence mechanisms [28].

**Figure 2:**
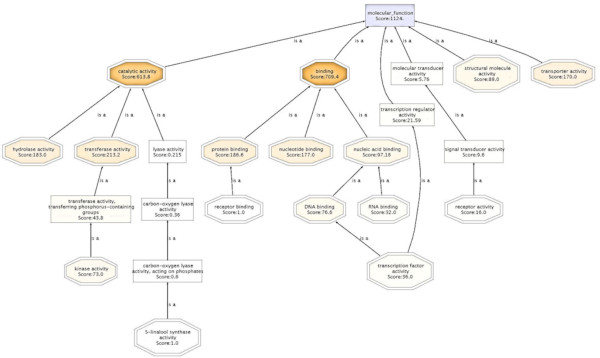
**A Directed Acyclic Graph (DAG) visualizing the hierarchical structure of the Gene Ontology (GO) in inoculated kumquat leaves**. Children that represent a more specific instance of a parent term have 'is a' relationship to the parent. The darker the color of the node the more number of Blast hits and the higher annotation score it has. All nodes contain the hit annotation scores in numbers.

**Figure 3:**
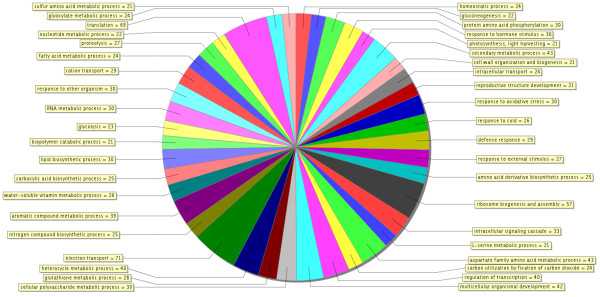
**A multilevel pie chart showing the distribution of probes on the chip**. Biological processes within all of the lowest nodes with the given number of sequences or score value plot jointly with an e- value cutoff (e-06).

Gene ontology analysis provided an extremely informative snapshot of the Xcc/kumquat interaction. The hierarchical structure for the gene ontology of a group of sequences can be visualized as a tree by means of directed acyclic graphs (DAG) [33]. For instance, the molecular functions of the network implicated in the kumquat response to Xcc infection is illustrated in the DAG presented in Figure 2. The graph demonstrates a tree controlled by the Seq filter that organizes the number of nodes to be displayed. Seq is the number of different sequences annotated at the child GO term. On the whole, the biological meaning for different sequences in the data set was best illustrated in terms of three GO gene categories; the biological processes (Figure 3) underlying molecular functions (Additional file 5), and the cellular compartments where proteins were localized (Additional file 6).

### Kumquat transcriptional changes in response to Xcc infection

**Figure 4:**
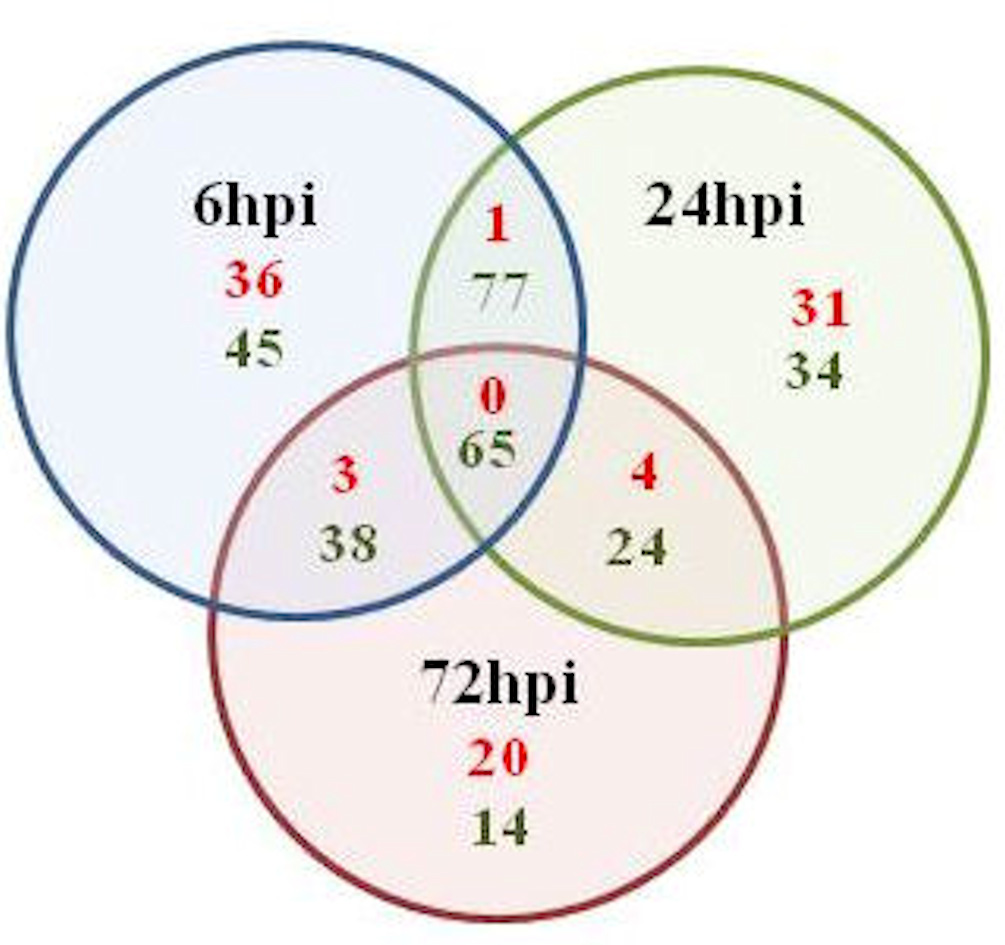
**Venn diagram demonstrating the number of up-regulated genes (numbers in red) vs the down-regulated (numbers in green) subsequent to Xcc inoculation**. Results were based on the mean inductions of six experimental replicates. Genes with M-values>0.5 (1.5 fold) were considered up-regulated while M-values<0 were considered down-regulated.

An important aspect of the data was that, for many genes, transcript abundance varied over time points, and a number of genes were only up- or down-regulated at one or two time points (Figure 4). Two approaches were used to identify patterns of gene expression. First; the ASCA-gene analysis methodology revealed that most of the total variability in the data was related to time-associated changes [34]. According to ASCA, 289 probes were selected as differentially expressed, 172 of which were at statistically significant levels (Additional file 7). Moreover, the time-associated variation could be divided into two main variability patterns. One pattern (accounting for 20% of the variation) represented genes whose expression levels changed significantly at 24 hpi from their levels at 6 hpi and then recovered to values similar to the starting values (or to even greater values in the opposite direction) at 72 hpi. However, the major pattern (80% of the time-associated variation) indicated a strong gene expression change between 6 and 24 hpi followed by preservation of expression levels at 72 hpi. This indicates that the strongest response to infection occurred at 6 to 24 hpi, and the majority of genes maintained their change for up to 3 days with a smaller percentage reverting to initial values.

**Figure 5:**
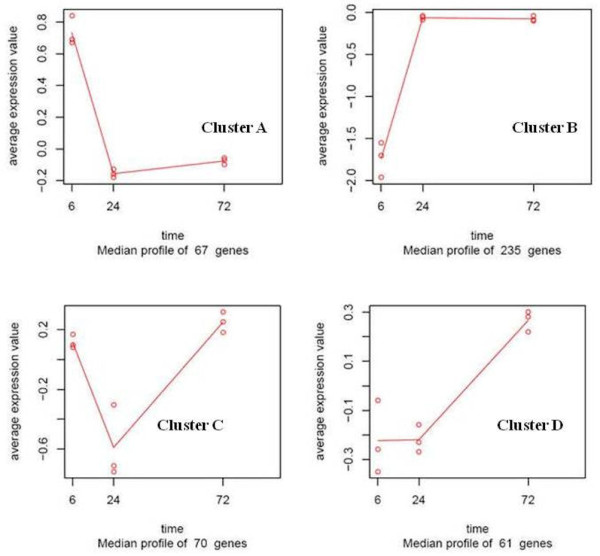
**Cluster patterns**. The overall average gene expression profiles for genes from different functional clusters at each time-point.

The second approach, maSigPro analysis, indicated that 317 probes were differentially expressed throughout time, (adjusted p value < 0.05 and R^2 ^of the model fit >= 0.6; Additional file 8). The results of both approaches were combined into 433 probes that were then filtered using more stringent conditions to provide a unique result. The union rather than the intersection of the two approaches was taken because the two methods reveal different aspects of the data and are thus complementary. The ASCA-gene methodology focuses on shared gene expression changes to find important genes, while maSigPro treats genes independently and evaluates significant time dependent-changes. Although ASCA-gene methodology may miss some genes whose expression pattern is rare but significant, these will be captured by the gene-wise maSigPro approach. Alternatively, maSigPro can miss genes with less pronounced changes, which can be recovered by ASCA-gene if their profile is abundant within the dataset. The use of both approaches together resulted in the identification of 437 differentially expressed genes 312 of which with acceptable p-values that could be divided into 4 clusters according to their expression patterns (Figure 5, Additional file 9). The criterion for this division is as follows. From the ASCA analysis we obtained the main patterns of variation: Cluster pattern A indicates a strong change in expression between 6 hpi and 24 hpi, which is then maintained at time 72 hpi. Cluster B pattern is comprised of genes differentially expressed at 6 hpi as compared to either 24 hpi or 72 hpi. For each pattern, the correlation of the mean value of each gene at each time point with the profiles indicated by ASCA-gene was calculated; subsequently genes were divided into 4 clusters depending on whether expression levels changed in positive or negative directions. In this analysis, genes cannot be classified simply as induced or repressed, because this depends on the time points considered; for example, genes in cluster pattern C are repressed at 24 hpi and then induced at 72 hpi.

### Functional categorization of transcripts underlines key elements in kumquat response to Xcc infection

Based on the assumption that altered gene profiles during plant-microbe interactions can be correlated with symptoms, gene ontology and annotation, we believe that Xcc represents a typical example of how the bacterial pathogen can manipulate the host systems in its favour as elucidated previously in different studies [3,4,11,35,36]. Information on all of the specific transcripts discussed in the subsequent paragraphs is given in Table 1. Cernadas *et al*. inoculated 'Pera' sweet orange with either Xcc, which causes typical canker symptoms on this citrus type, or *Xanthomonas axonopodis *pv. *aurantifolii *pathotype C (Xaa), which only produces symptoms on Mexican lime, followed by a detailed transcriptional analysis for the sweet orange plants [36]. Although the analyses done in that study cannot be directly compared with our study because of differences in methodology, some generalizations are noted below.

**Table 1:** Functional categorization of cDNAs identified from microarray analysis.

Putative Function	Cluster	P-Value	ID	M-Value		
				
				6 hpi	24 hpi	72 hpi
**OXIDATIVE BURST/STRESS, APOPTOSIS**						

*Thioredoxin f	A	0.093	KLLFI3-F09	**+0.093**	**+0.037**	**+0.913**

*Peroxidase	**C**	0.046	KLLRI2-F09	**-2.370**	**-0.280**	**-0.380**

*Class III peroxidase	**B**	0.018	KSLFI1-H03	**+1.643**	**+0.323**	**+1.053**

**Glycosyl transferase-like** **protein**	**D**	0.004	KSLFI7-F12	**-2.237**	**+0.103**	**-0.243**

*Glutathione peroxidase	**D**	0.024	KSLFI1-B02	**-2.143**	**-0.027**	**-0.287**

*Formate dehydrogenase	**B**	0.041	KLLRI2-G05	**+0.930**	**-0.447**	**+0.173**

*CuZn-superoxide dismutase	**B**	0.035	KLLFI3-A03	**+0.523**	**-0.267**	**-0.086**

*Protease inhibitor	**B**	0.015	KLLFI2-D02	**-1.987**	**+0.653**	**+0.510**

*lon protease homologue	**D**	0.074	KSLFIV1-H05	**+1.220**	**-0.607**	**+0.240**

*Dehydroascorbate **reductase **	**C**	0.012	KSLR1-F02	**-2.033**	**-0.040**	**-0.223**

*Glutathione peroxidase	**C**	0.021	KLLFII3-G07	**0.267**	**-0.237**	**-0.247**

*Ubiquitin-conjugating enzyme ubc7	**B**	0.015	CSL1-A02	**-0.163**	**-0.390**	**+0.320**

**Catalase (EC 1.11.1.6)CAT-2 **	**C**	0.041	KLLFI1-F11	**-1.343**	**-0.207**	**-0.253**

*Amine oxidase	**A**	0.035	KSLFI3-G05	**-0.553**	**-0.720**	**+0.493**

**Hydroperoxide lyase**	**B**	0.012	CSL2F2-A01	**-2.963**	**-0.427**	**-0.167**

**Benzoic acid salicylic acid methyltransferase**	**C**	0.034	KLLRI2-C03	**+0.103**	**-0.233**	**-0.263**

*1-aminocyclopropane-1- **carboxylate oxidase**	**B**	0.008	KSLFI7-H12	**-1.993**	**+0.183**	**-0.310**

**PHOTOSYNTHESIS**						

**Chlorophyll ab binding protein**	**B**	0.006	KLLFIII3-A06	**-2.057**	**+0.217**	**+0.563**

**Chloroplast photosystemII 22kda**	**B**	0.15	KLLFIII3-E08	**-0.717**	**+0.433**	**-0.050**

**DEFENCE**						

*Pathogenesis-related protein 1a	**A**	0.054	KSLFI3-H10	**-1.803**	**-0.077**	**-0.407**

*SABP2	**B**	0.008	KLLRI2-G01	**+0.787**	**-0.093**	**-0.317**

*Beta-1,3-glucanase	**B**	0.024	KSLFII1-C07	**-1.91**	**-0.760**	**-0.167**

**Phenylalanine-ammonia lyase**	**D**	0.016	KSLFI4-F04	**-2.58**	**-0.013**	**-0.147**

**Pathogenesis-related protein 4-1**	**A**	0.032	KLLFII2-G01	**+0.07**	**-0.210**	**-0.353**

*Class IV chitinase	**C**	0.626	KLLRI2-D05	**+0.01**	**-0.3**	**-0.28**

*NDR1 homologue	**C**	0.132	KLLFII2-E03	**-0.91**	**-0.74**	**-0.04**

*Trypsin inhibitor	**A**	0.011	KSLFIII1-H12	**-0.40**	**+1.05**	**+0.303**

*Trypsin inhibitor	**B**	0.008	KLLFIII3-F03	**-3.20**	**+0.197**	**-0.490**

*HSR203J-like protein	**C**	0.003	KSLFI3-C10	**+0.073**	**-0.09**	**-0.303**

*DND1 [Arabidopsis **thaliana] **	**D**	0.074	KLLRI2-B05	**-0.203**	**-0.580**	**+0.310**

*Bax inhibitor-1	**C**	0.01	KLLFIII2-E02	**+0.233**	**+0.083**	**+0.06**

*Latex-abundant (caspase **-like) **	**B**	0.090	KLLRI2-A12	**+0.230**	**+0.173**	**+0.09**

*Zinc finger protein	**B**	0.017	KSLFI6-C10	**+2.387**	**+0.000**	**+0.663**

M-value is the base two logarithm of the ratio between the background-subtracted foreground intensity measured in the red and the green channels.

These ESTs were identified as having a cy5 cy3 ratio > ± 1.5 for four out of six spots on the microarrays.

- Putative function determined with the Gene ontology sequence description

- Cluster: The cluster to which the putative gene belongs according to Blast2 GO functional analysis.

- P-value associated to the statistical analysis for differential expression adjusted for multiple comparisons.

- ID: Assigned at selection.

- M-Value: A metric for comparing a gene's mRNA-expression level between two distinct experimental conditions; in this case mock inoculated vs Xcc inoculated.

- (-) means down-regulated where M-value < 0 while (+) is up-regulated where M-value > 0

Table 1. includes (*) genes that are discussed in the text.

While other sequences that might not be mentioned in the text but show interesting gene expression profiles.

**Figure 6:**
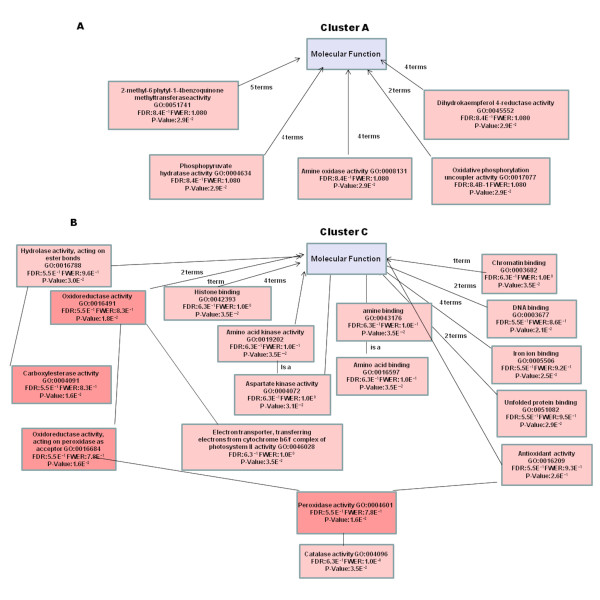
**Blast2GO directed acyclic graph showing "molecular function" after Xcc inoculation among transcripts representing the enriched functional categories (P < 0.25)**. (A) Cluster A. (B) Cluster C.

**Figure 7:**
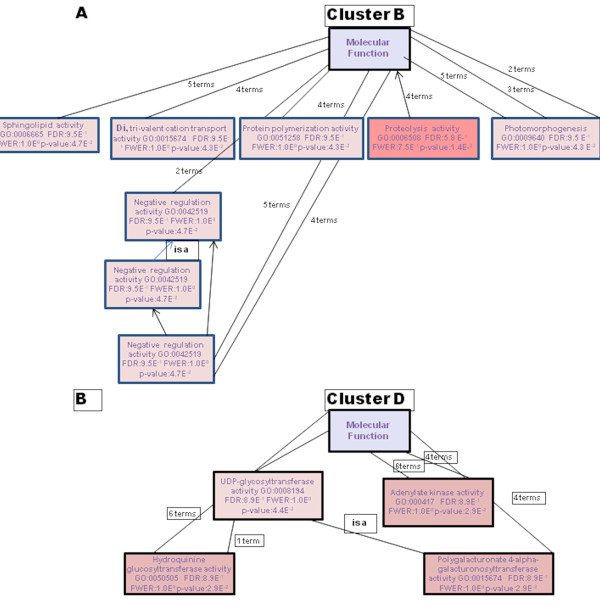
**Blast2GO directed acyclic graph showing "molecular function" (P < 0.25) among transcripts induced at 24hpi**. (A) Cluster B. (B) Cluster D.

The distribution of functions within the significantly expressed genes in Xcc infected kumquats indicates that the highest number of transcripts (~30%) was associated with response to stress, electron transport, and/or oxidative stress (as shown in Figure 3), an indication of an early regulatory changes in the plant immune system by Xcc. Earlier studies, such as that of Cernadas *et al.*, have come to the same perception [36]. Each identified cluster was subjected to functional analysis by either studying the distribution of GO terms or performing enrichment analysis to see if there were functional categories that were significantly represented. A total of 137 genes, which makes up more than 30% of the genes that were significantly expressed, were down-regulated in the interval between 6 hpi and 24 hpi,. Most of them were grouped in Clusters A and C (Figure 6). The expression levels of the genes in both of these clusters reached a minimal expression level at 24 hpi followed by either a minor (Cluster A) or major (Cluster C) recovery by 72 hpi (Table 1). For instance, the expression of the thioredoxin f gene homologue (KLLFI3-F09) that belongs to cluster A reached its maximum level of expression (+1.8 fold) by 72 hpi after slight decrease at 24 hpi.The lipoxygenase gene homologue (KSLFII1-F07) that belongs to cluster C was 1.5 fold down-regulated at 6 hpi followed by a 3 fold increase in expression when compared to the its expression level at 6 hpi sample. Genes in Cluster A and C were frequently related to oxidative stress response. Most of the activity for genes in these clusters is located in the mitochondria, the cell membrane and the chloroplast (Figure 6). Cluster B was the largest cluster and included 235 genes with up-regulated expression levels between 6 hpi to 24 hpi followed by sustained expression until 72 hpi (Figure 5, Figure 7A). Cluster D contained 61 members that had a low steady expression up to 24 hpi, and were subsequently upregulated (Figure 5, Figure 7B). This cluster includes genes, such as the glycosyltransferase-like gene (KSLFI7-F12), that mediate the transfer of glycosyl residues from activated nucleotide sugars to acceptor molecules (aglycones), a key mechanism in determining the diversity, activity and chemical complexity of plant natural products. In plants, UGTs (uridine diphosphate sugar glycosyl transferases) generally use UDP-glucose and occasionally UDP-xylose for glucosylation of phenylpropanoid aglycones. Albrecht and Bowman [37] proposed using UGTs and other glycosyltransferases as prospective genetic engineering candidates due to their important role in resistance and tolerance to citrus tristeza virus (CTV) as well as citrus huanglongbing (HLB) in trifoliate oranges (*Poncirus trifoliata *L. Raf.). Phenolics are mainly synthesized in plants via the phenylpropanoid pathway and are incorporated into many important compounds including plant hormones, secondary metabolites involved in stress, defence responses, and xenobiotics such as herbicides [38]. In addition, phenylpropanoid pathway intermediates, for example p-coumaric acid, caffeic acid, ferulic acid and sinapic acid, and pathway derivatives, including flavonoid aglycones and glycosides, exhibit antimicrobial activity [39,40].

### Kumquat transcriptional changes in response to Xcc infection

#### ROS vs ROS scavenging

In order to maintain homeostasis and overcome the damaging effects of ROS (reactive oxygen species), a balance between SODs (superoxide dismutase) and the different H_2_O_2_-scavenging enzymes is considered to be critical in determining the levels of O_2_^- ^and H_2_O_2 _in plant cells [41,42]. Accordingly, there is a constant interplay between the antioxidant state and processes generating ROS. ROS are produced in chloroplasts, peroxisomes, and mitochondria in response to biotic as well as abiotic stresses [43,44]. Accordingly, the expression of different enzymes that produce ROS were evidently stringently controlled and coordinated during the kumquat/Xcc interaction. For instance, while formate dehydrogenase (FDH; KLLRI2-G05), a mitochondrial NAD dependent enzyme, was 1.5 fold upregulated by 6 hpi, amine oxidase (KSLFI3-G05) that contributes to the synthesis of H_2_O_2 _and secondary metabolites was downregulated by 1.5 and 1.6 fold at 6 and 24 hpi respectively in response to Xcc challenge (Table 1). Concurrently, Xcc-inoculated kumquat plants overexpressed genes related to ROS scavenging to restrict damage to the inoculated parts of the plant, in this case the leaves. For instance, CuZnSOD (KLLF13-A03) expression in kumquats was increased ~1.5 fold at 6 hpi and was stabilized at 24 hpi and 72 dpi (Table 1). The same phenomenon was observed previously in tomato infected with *Botrytis cinerea*, a sign of increased ROS production by the host as part of the defence response to infection [45]. Furthermore, while the expression of some of the genes linked to protease inhibitors and endopeptidase activities such as protease inhibitor homologue (KLLFI2-D02) was suppressed by the bacteria, other serine-type endopeptidase inhibitors such as an ATP-dependent ion protease (KSLFIV1-H05) was >2 fold up-regulated as early as 6 hpi subsequent to Xcc inoculation. In the same context, the redox coupling ascorbate-glutathione cycle, known to be responsible for peroxide detoxification [46], was repressed by 6 hpi in the kumquat dataset; examples include dehydroascorbate reductase (KSLRI1-F02) and glutathione peroxidase (KLLFII3-G07). Ascorbate and glutathione are non-enzymatic antioxidant molecules that have a role in other cycles, including those that synthesize and in some cases modulate flavonoids, alkaloids, phenolic compounds, α-tocopherol and carotenoids, all of which contribute in scavenging ROS [47]. Dehydroascorbate reductase activity is indispensible when the ascorbate peroxidase (APX) levels are higher than normal under certain conditions to ensure preservation of the reduced form of ascorbate. Both proteins in addition to certain types of trypsin inhibitors might also catalyze a plant response [48]. A similar study to investigate the Xanthomonas-grapefruit compatible interaction might present a platform to compare gene expression profiles of some genes of interest in both plants.

Accumulating evidence indicates that protein ubiquitination and degradation, last steps in protein turnover, are involved in plant defence responses. A number of recent studies have investigated a possible role of U-box E3 ubiquitin ligases in PTI (PAMPS-triggered immunity), ETI (effector-triggered immunity), as well as plant cell death and defence [49,50]. In the present study, 6 ubiquitination pathway-related genes, for example ubiquitin-conjugating enzyme ucb7 (CSL1-A02), were isolated in the kumquat forward subtracted libraries; more investigation of their expression levels after infection will follow. Other induced genes that are involved in the proteolysis process are present in clusters A, B and C.

#### Genes involved in photosynthesis

A distinct down-regulation in the expression of ribulose1,5-bisphosphate carboxylase/oxygenase at 6 hpi, followed by an increase in expression that reaches maximum expression at 24 hpi, was observed in the microarray dataset (Rubisco small and large subunits; for example KLLFIII3-G09 and KSLRII2-F01) (Table 1). Rubisco, the most abundant protein in leaves, is the main source of energy production in plant cells. A decrease in photosynthesis was previously shown in *Arabidopsis *leaves as early as 3 h after challenge with the *P. syringae *avirulent strain, while after 48 h the rate of photosynthesis was lower with the virulent strain [51]. Most of the photosynthetic machinery in challenged kumquat leaves was repressed at 6 hpi, including chlorophyll A/B binding protein (KLLFIII3-A06 and KLLFIII3-E08). Three photosynthesis-related genes were differentially down-regulated during the first 24 hours. In Pto-mediated resistance, 30 photosynthesis-related genes and 12 genes encoding chloroplast-associated proteins were suppressed [52]. These results show that plants reduce photosynthetic potential to induce HR following pathogen attack. Further, Quirino et al. [53] suggested that HR and senescence are two programs that involve biochemical similarities as well as an overlap. The research reinforced the idea of a connection between defence response and senescence. Evidently, the down-regulation of genes involved in photosynthesis during the Xcc/kumquat interaction represents a cost for the plant fitness where energy resources were redirected to defence response.

#### Cell wall remodelling

Xcc inoculation of kumquat was followed by the down-regulation of various genes related to cell wall remodeling and rapid expansion such as endoglucanases. The expression level of a kumquat homologue of this wall loosening protein (KLLFI2-C10) was insignificant. On the other hand, genes related to cell wall reorganization, for example xyloglucan endotransglycosylase/hydrolase (XET,-- an enzyme involved in cell wall elongation and restructuring), were significantly up-regulated by 24 hpi (KSLFIII1-H08). In 'Pera' sweet orange, a major difference in the response to inoculation of the two bacterial strains was that Xcc strongly upregulated several cell wall remodelling enzymes, while Xaa upregulated genes related to endoglucanase inhitors and lignin biosynthesis. A phenomenon that we observed in kumquat plants is the development of a few minute necrotic flecks on the leaves when inoculated with low concentrations of the bacterium (Xcc). Neither leaf abscission nor water soaked lesions were observed on the leaves later under our conditions. It is also worth mentioning that although Cernadas et al. used a relatively high concentration of Xaa (OD600 nm = 0.6, ~double what we used for Xcc with kumquat) only pustles were recorded in sweet orange inoculated with Xaa that were not followed by necrosis [24]. Using light microscopy, we have previously shown mesophyl collapse in kumquat leaves which was followed by leaf abscission 72 hrs post inoculation with Xcc. Alternatively, grapefruit mesophyl cells from inoculated leaves showed enlargement (hypertrophy) and division (hyperplasia) followed by raised circular lesions that became raised and developed into white or yellow spongy pustules. These pustules then darkened and thickened into brown corky canker lesions [24]. Pustule formation and hypertrophy were linked previously to the PthA effector in *Nicotiana benthamiana *[35]. Alternatively, accumulation of the tomato *XTH *(xyloglucan endotransglucosylase/hydrolase *LeXTH1*) protein 6 hours after attachment of the parasite has provided evidence for a role of *XTH *in defence reactions associated with the incompatible tomato-*Cuscuta *interaction as was presented in Albert et al. [54].

#### Resistance genes and related proteins

Most of the differentially expressed genes in the kumquat/Xcc interaction have also been identified in plant-insect interactions [55], non-host resistance [56] and R-gene mediated resistance [52], suggesting a high level of convergence between different types of resistance mechanisms. The expression levels of some homologues to the PR3 (endochitinase) (KFII1-B11) and ß-galactosidase (BG1) (KRI2-D05) genes were found to be modestly activated; 2 and 1 fold up-regulated respectively 6 hpi, although both genes were down-regulated by 24 hpi as shown by the microarray and the qRT-PCR (quantitative real-time PCR) data (Table 2, Additional File 10). Chitinase expression is a plant defence strategy typically used against wall components of fungi and insects [57]. According to the qRT-PCR, the kumquat chitinase gene was >2-fold up-regulated by 6 hpi, after which it was suppressed at 24 hpi, and then its expression increased. Other studies have also reported the induction of chitinases in response to bacterial pathogens but their function is not well known [58].

**Table 2:** qRT-PCR analysis of genes expressed in response to Xcc inoculation a (5 × 10^8^cfu/ml) concentration of the Miami strain X04-59.

*Gene ID*	*Function*	*Microarray* *(Fold Change)*				*Real-time PCR* *(Fold Change)*			
		6 h	24 h	72 h	0 t	6 h	24 h	72 h	120 h

KLLFII2-C05	Basic leucine zipper transcription factor	-1.04	+1.06	-1.21	1	-0.53	+1.13	+1.57	+0.26
KLLRI2-D05	Class I chitinase (CHI1)	+1.01	-1.23	-1.21	1	+2.9	-0.16	9.21	-4.53
CSL1-D05	A disease resistance leucine-rich repeat protein	+1.06	+2.04	+1.08	1	+3.09	+7.73	+1.66	+0.19
KLLRI2-H10	Receptor-like serine threonine kinase	+1.62	-1.02	-1.19	1	+2.65	+0.51	+0.53	+0.47
KLLFII1-B11	A putative beta-galactosidase BG1	-1.13	-1.24	+1.01	1	-1.24	-0.10	+0.91	+0.19
CSL2-A02	A mitogen-activated protein kinase 3	+1.02	-1.11	+1.16	1	+5.66	-14.1	+1.22	+0.58

Interestingly, the expression levels of the kumquat PR1 (Pathogenesis -related gene1) gene homologue, normally a marker of salicylic acid-induced systemic acquired resistance (SAR) that is usually up-regulated after pathogen infections, was lower in the infected samples as early as 6 hpi compared to control mock inoculated samples (KLLFII2-A04) (according to the microarray results and non-published data). The region upstream of the PR1 promoter, W-box sequences, was shown previously to act as a negative cis-acting element in the expression of defence related genes [59]. This implies a different basal defence response and SAR regulation mechanism from that of Arabidopsis and other dicotyledonous plants in kumquat after Xcc infection. PR genes that were identified in citrus vary in their responses to different pathogens as shown in this study and others where variation in expression levels of different members of the PR gene family was dependent on the nature of different elicitors [60-62]. The biological activity of a large majority of PR genes in plants during biotic stress is yet to be revealed. More interestingly, according to the microarray results, expression levels of a number of homologues to other defence related genes such as the NDR1 gene (KLLFII2-E03) were also repressed 24 hpi after Xcc infection.

LRR proteins are known to be a part of the early signal transduction cascade involved in the recognition of pathogen Avr products [63]. Sequences for a number of homologues known to be part of different hormonal defence pathways (for instance transcription factors, receptor like and receptor-like kinases) were found to be differentially expressed in kumquat after Xcc inoculation.

#### Key molecular features of kumquat PCD

A number of genes homologous to known resistant response-specific genes were expressed in the kumquat transcriptome concurrently following Xcc inoculation, listed and discussed below:

(i) KSLFI3-C10 is homologous to *hsr203J *, a carboxylesterase (CXE) gene implicated previously in the incompatible interactions between tobacco and the bacterial pathogen *Ralstonia solanacearum*. Its promoter is highly, rapidly, and specifically activated in response to HR inducing bacterial inoculation, does not respond to various stress conditions, and is strongly dependent on *hrp *(hypersensitive response and pathogenicity) genes of the pathogenic bacterium [64]. It has been proposed that its expression should be a useful marker for programmed cell death occurring in response to diverse pathogens.

(ii) KLLRI2-B05 shares homology with DND1 (DEFENCE NO DEATH 1), which encodes a cyclic nucleotide-gated ion channel that allows passage of Ca2+, K+ and other cations. The *Arabidopsis thaliana dnd1 *mutant failed to produce HR cell death in response to an avirulent pathogen infection [65].

(iii) During programmed cell death or apoptosis cytochrome c is released to the cytoplasm from the intermembrane space of the mitochondrion [66,67]. Once in the cytoplasm, it activates caspases (cysteine aspartate-specific proteases), killer proteins that dismantle the cell [68]. Two key proteins known to be core components of the apoptic machinery in animals, caspase and *Bax-Inhibitor1 *gene homologues, were identified in our dataset. According to our data, a homologue that has caspase activity KLLRI2_A12 was slightly up-regulated by 6 hpi in kumquat challenged leaves. Bax is a member of the Bcl2 family that plays a regulatory role preventing apoptosis by inhibiting adapters needed for the activation of caspases [54]. A kumquat homologue of the *Bax-Inhibitor1 *gene (KLLFIII2-E06) was shown to be slightly up regulated 6 hpi in response to Xcc challenge as was previously shown with *Arabidopsis thaliana *Bax Inhibitor-1 (*AtBI-1*), isolated during a differential screen of plants challenged with the phytopathogen *Pseudomonas syringae *[69]. In the same context, Bax inhibitor has been shown to trigger cytochrome c release from mitochondria both *in vitro *and *in vivo *in animals.

(iv) Endopeptidase inhibitors are often part of an inducible, jasmonic acid associated defence pathway that accumulates upon wounding, pathogen, or herbivore damage in leaves [26]. The antagonistic interaction between proteases and endopeptidase inhibitors is considered to be a cell death control mechanism [70]. Li *et al., 2008 *demonstrated that a serine protease (Kunitz trypsin) inhibitor (KTI1) of Arabidopsis is involved in modulating PCD in plant-pathogen interactions [71]. RNAi silencing of the AtKTI1 gene resulted in enhanced lesion development after infiltration of leaf tissue with the PCD-eliciting fungal toxin fumonisin B1 (FB1) or the avirulent bacterial pathogen *Pseudomonas syringae *pv tomato DC3000 carrying avrB (Pst avrB). Trypsin inhibitor (KSLFIII1-H12 and KLLFIII3-F03) and a miraculin serine type endopeptidase inhibitor (FI2-A05) sequences were found in original early subtraction libraries representing transcripts expressed during early infection (30 min.pi-24 hpi). While KLLFIII3-F03 (trypsin homologue) gene expression was significantly suppressed 6 hpi, the expression of KSLIII1-H12 was slightly suppressed and then 2 fold upregulated 24 hpi. Further analysis should be done to study the difference between the mechanism(s) of action of these two genes.

#### Suppression of defence responses

A very evident down-regulation of a considerable number of genes was recorded by 6 hpi which may be caused by defence suppression imposed by Xcc effectors (Clusters A and C; Figure 6A and 6B). It has been shown previously that Xcc exploits the Type III secretion system (T3SS) to inject different effector proteins into citrus plants in order to avoid host recognition and subsequently MAMPS/PAMP-triggered immunity. The bacterial effector proteins suppress plant defences including basal defence, gene-for-gene resistance, and nonhost resistance. There was no accumulation of any SAR gene transcripts including PR1, a marker for enhanced defence; in addition some other key elements in the SA defence pathway were suppressed. On the other hand, the S-adenosyl-l-methionine:benzoic acid salicylic acid carboxyl methyltransferase gene (KLLRI2-C03) was at least 1-fold upregulated in kumquat leaves by 6 hpi in response to Xcc inoculation; the gene is known to play a role in plant defence responses [72]. In addition, the SA-binding protein 2 (SABP2 KLLRI2-G01), a lipase protein that belongs to the hydrolase super family, was found to be up-regulated at 6 hpi by at least 2 fold; the gene was previously found to be required for the plant immune response in tobacco [73].

#### Realtime Quantitative Polymerase Chain Reaction Validation

Validation of the presented microarray dataset was carried out using TaqMan gene expression assay for a number of homologues on the array. Genes that were implicated in plant defence including basic leucine zipper transcription factor (KLLFII2-C05), a putative chitinase protein (CHI1)(KLLRI2-D05), a putative disease resistance leucine rich protein (CSL1-D05), a receptor like protein kinase (KLLRI2-H10), a beta galactosidase like protein (KLLFII1-B11) and a putative mitogen-activated protein (CSL2-A02) were selected for validation using q RT-PCR. As summarized in Table 2 and Additional file 10, the qRT-PCR data correlated with the microarray results confirming the up-or down-regulation of all analyzed genes although as expected the qRT-PCR was more sensitive.

## Conclusions

In this study, a *F. margarita *custom microarray representing 1024 unigenes was used to study the response to inoculation with *X. axonopodis *pv. *citri*. A very distinct though delayed HR was observed in Xcc-inoculated kumquat plants where initially the bacterium grew exponentially, followed by a sudden leaf tissue collapse (necrosis with no canker lesions) 2-5 days after inoculation [24]. A comparable delayed HR was observed in tomato resistance response to race T3 mediated by AvrXv3 effector and RxvT3 R protein [74]. The current kumquat analysis allowed simultaneous investigation of the expression of more than one group of genes known to be linked to more than one biological process and cellular compartment in relation to the HR caused by Xcc infection. A large number of genes were found to be differentially expressed after infection. Most of the genes involved in defence mechanisms in kumquat appear to be associated with the phenomena that precede the HR including oxidative burst, protein degradation, and regulation of photosynthesis as well as the production of ROS that is associated with the oxidative burst. One very distinct observation was that some of the defence genes such as PR1 and NDR1 were down-regulated in kumquats in response to Xcc inoculation as early as 6 hpi, a phenomenon currently under further examination. What clearly appears to be a resistant response and a drastic decrease in the bacterial population, in addition to the activation of genes involved in ROS production as well as and programmed cell death, seems to be a common mechanism that is pursued by more than one citrus bacterial pathogen with no associated-resistance genes yet identified [24,36]. Future work will compare differences in gene response in both resistant and susceptible citrus types.

## Methods

### Plant material and inoculation with bacteria

*Fortunella margarita *(Lour.) Swingle (Nagami kumquat) plants were used in all of the experiments described in this study. Plants were approximately 2 years old at the time of the experiment and were maintained in the quarantine greenhouse facility at the Division of Plant Industry, Florida Department of Agriculture (Gainesville, FL, USA) under controlled conditions. Leaves from a set of six kumquat plants were infiltrated with bacterial cultures according to Lund et al. [75]. The bacterial strain used was *Xanthomonas citri *subsp. *citri *A; Miami X04-59 (Xcc). The inoculum was adjusted to 5 × 10^8 ^cfu/ml. A similar set of plants was mock-inoculated using sterile tap water as controls. Leaves from the two sets of plants were used in subsequent experiments.

### Microarray platform

The kumquat microarray chip was developed and printed at the University of Florida (Gainesville, FL, USA). The array included ESTs chosen from 4 previously constructed Nagami kumquat forward and reverse leaf subtraction cDNA libraries. The cDNA libraries were constructed using RNA extracted from leaf tissue collected at different intervals post inoculation with Xcc (see below) and pooled into early and late library sets to capture a wide spectrum of differentially expressed transcripts [24]. Random DNA sequencing was performed from 5'and 3' ends of randomly selected clones using universal primers, generating sequence information from 2788 and 1655 clones from the early and late leaf subtraction libraries respectively.

The initial dataset was reduced to a total of 2304 transcripts that were selected according to sequence alignment similarities with proteins in the Genbank database. Sequences were selected based on quality and length. The dataset included 2254 kumquat ESTs comprising 738 contigs and 1516 singletons, in addition to 50 cDNA control elements. Each probe was printed in 3 locations on the array using the Omnigrid Microarrayer (Gene Machines, San Carlos, CA, USA) so that all clones had 3 technical replicates on each slide, generating a total of 6912 spots. Post-printing slide processing was performed as described in Heller et al. (1997) [76] with some modifications. In brief, a combination of sequential baking and UV crosslinking was implemented where slides were baked for 80 min at 80°C in a drying oven without vacuum. The slides were then washed twice in 0.1% SDS for 5 minutes each to remove any unbound DNA.

### Experimental plan

To identify genes that are considered to be differentially expressed in kumquat, a time-course experiment was designed utilizing the kumquat/Xcc pathosystem. Six independent Xcc or mock-inoculated kumquat plants were used; each plant was considered an independent biological replicate. Since citrus canker is a non-systemic disease, 6-10 leaves per treated kumquat plant were independently infiltrated using 5 × 10^8 ^cfu/ml Xcc. All RNA samples isolated from healthy mock- or Xcc inoculated (infected) leaves were processed independently. It is unlikely that differential gene expression observed was caused by the pressure infiltration inoculation method used, since this factor was normalized by treating the mock inoculated plants in the exact same way as infected plants.

Individual leaves were harvested from the inoculated and mock-inoculated plants at specific time-points post-inoculation (pi) according to designated conditions for each experiment; there were 3 time points for the microarray experiment and 5 for subsequent real-time PCR assays. For the microarray experiment, the three time points (6 hpi, 24 hpi, and 72 hpi) were chosen based upon the internal bacterial populations previously detected at these times following inoculation and the knowledge that kumquat leaves abscised 3-5 days after inoculation. In addition, previous experiments revealed that there were some transcripts differentially expressed as early as 30 min post-inoculation with Xcc. Finally, the RNA yield and the abundance of cellular transcripts decreased as the leaves approached total PCD, as has been shown previously by others [53]. Time points of 0 and 120 hpi were added for the real time PCR assays. The healthy mock-inoculated and Xcc-inoculated leaf samples were immediately frozen in liquid nitrogen. For each respective time point, total RNA was extracted using RNeasy columns (QIAGEN, Valencia, CA, U.S.A.) according to the manufacturer's protocol. RNA purity, concentration and quality were assessed using a spectrophotometer and a BioAnalyzer 2100 (Agilent Technologies, Palo Alto, CA).

### Fluorescent probe, hybridization, and scanning

Prior to slide hybridization with probe, slides were pre-hybridized in a solution containing 5× SSC, 0.1% SDS and 1% bovine serum albumin at 42°C for 45 min to eliminate nonspecific binding of the probe to the slide. Slides were washed using MilliQ RNase free water, then isopropanol, once each, and air-dried. Slides were maintained at the hybridization temperature until loaded with probe. cDNA labeling was performed using the Genisphere (Hatfield, PA, USA) 3DNA Array50^® ^Expression Array Detection Kit according to the manufacturer's protocol for total RNA. For each time-point 125 μg of DNA-free total RNA isolated from an independent plant (biological replicate) per slide was reverse transcribed for each of the mock inoculated and the infected leaf samples separately using Ambion reverse transcriptase (Ambion; LaJolla, CA) in the presence of Genisphere dT primers. Two-step hybridization was performed as follows. The first hybridization, carried out at 48°C overnight, contained 10 μl of the concentrated cDNA (heat denatured probe) made using either the Cy5-RT primer capture sequence or the Cy3-RT primer capture sequence, in Genisphere 2× formamide-based hybridization buffer. Three successive post hybridization washes were performed, first in 2X SSC, 0.2% SDS at 55°C for 10 min, then 2× SSC for 10 min and finally in 0.2× SSC for 10 min at room temperature. The second hybridization (for addition of the dye) was performed using 2.5 μl of either Cy3 or Cy5 dendrimer, 2 μl of high-end differential buffer and 58 μl of hybridization buffer. For each time point, three mock-inoculated samples from three individual plants were labeled with Cy-3 and the Xcc infected samples were labeled with Cy-5 and both were hybridized to the same array. Post-hybridization washes were conducted as performed earlier following the primary hybridization, with the addition of 0.1 ml dithiothreitol (DTT) into the first and second wash solutions to reduce oxidation of fluorescent dyes.

### cDNA microarray setup and quality control

Control measures such as the detection sensitivity level were determined using internal control probes and non-specific control elements. Human genomic DNA, the green fluorescent protein gene, and the lambda control template DNA fragment were included as negative controls. Additionally, cDNAs previously implicated in pathogen defence such as PR1 and NPR1 from *Arabidopsis*, NDR1 from citrus, were printed 3 times on the array to test the ability of the microarray method to detect changes in gene expression. These were considered to be specific positive controls. In addition, the microarray ratio for each gene analyzed was normalized against the microarray ratio obtained for 18 S.

### Transcriptome data analysis

Agilent's Feature Extraction Software (Agilent Corp., Palo Alto, CA) was used to analyze the microarray data. Data were uploaded into the statistical platform R [77] for statistical analysis and the Limma package was used for pre-processing. Data were Lowes transformed followed by scaling between arrays [78]. Two array slides were chosen for each time-point experiment based on the consistency of the signal across the replicates. The fold difference in expression was computed as: 2٨average ratio (2 to the power of the average ratio). cDNAs with an average ratio of 1.0 or higher were considered differentially expressed, which represents a 1.5 fold or higher difference in expression. Statistical analysis was performed using two different approaches. Time-dependent gene expression changes were analyzed by the maSigPro methodology [79]. Data were subsequently subjected to ASCA-gene analysis that combines ANOVA and multivariate methods to identify main and secondary patterns of gene expression associated with different experimental factors [34]. Statistical analysis identified a number of selected genes that were further grouped into clusters.

Functional information about the ESTs represented in the array was obtained by Blast2GO analysis using default parameters [29]. Blast2GO uses Blast and an elaborated annotation algorithm to assign Gene Ontology (GO), Enzyme Code and InterPro functional labels to a set of uncharacterized sequences [27]. The functional characterization of these clusters was done by applying the Functional Enrichment (FE) method included in Blast2GO which implements the Gossip algorithm. FE methods assess which functional categories are over-represented within a group of genes in relation to a broader list, in this case the whole kumquat array. Finally, the major induced transcriptional changes considered functional classes as a whole were studied with the PCA-maSigFun method [80]. This method combines Principal Component Analysis and maSigPro to characterize the "expression profiles" associated with cellular functions. Sequence data from this work have been deposited in the NCBI Genbank database libraries (http://www.ncbi.nlm.nih.gov/GenBank/index.html), using the BankIt dbEST database, and accession numbers were obtained. [Genbank: GW687757to GW690680]. (See Additional File 11).

### Quantitative Real-time Quantitative PCR

Kumquat leaves were infiltrated with Xcc (5 × 10^8 ^cfu per milliliter), then total RNA was isolated from inoculated leaves 0,6,24,72, 120 hpi for both the microarray and the quantitative real-time PCR experiment as previously stated in the 'experimental plan'.

Total RNA was isolated separately for each respective time point using the TRIzol reagent (Invitrogen, Carlsbad,CA, U.S.A.) according to manufacturer's instructions. RNA samples were further purified using the RNeasy Plant Mini Kit (Qiagen, Valencia, CA) including DNaseI TURBO DNase (Ambion, Austin, TX, U.S.A.) treatment according to the manufacturer's instructions. RNA quality and quantity was then assessed using microspectrophotometry (Nanodrop Technologies, Inc., Wilmington, DE). cDNA was synthesized from 1 μg of total RNA using Applied Biosystems (High Capacity cDNA Reverse Transcription Kit PN 4368813, 4374966). A TaqMan gene expression assay was then used to validate the transcript accumulation levels of a specific subset of genes from the kumquat microarrays. Reactions were performed in the ABI Prism7900 HT sequence detector (Applied Biosystems, Foster City, CA, U.S.A.). Primers for qRT-PCR were designed using the Primer Express 3.0 software (Applied Biosystems), and data were normalized using a Taqman ribosomal RNA control, in addition to the kumquat 18S ribosomal gene that served as an internal control where each real-time PCR reaction was done in parallel with the 18S primers. For internal controls, a number of genes, for example actin showed inconsistencies. The 18s surprisingly showed coherency throughout the interaction, and this was noticed during the microarrays and was subsequently confirmed with the Realtime PCR (RT-qPCR) 18S expression curve. qRT-PCR was carried out at 50°C for 2 minutes, 95°C for 10 minutes, followed by 40 cycles at 95°C for 15 seconds and 60°C for 1 minute.

## Authors' contributions

AK carried out the molecular genetic studies, participated in data analysis, and drafted the manuscript. AC carried out the B2GO statistical analysis supervised by JD. FG participated in the design of the study in addition to coordination between the authors and review of the manuscript. GM participated in its experimental design and reviewed the manuscript. All authors read and approved the final manuscript.

## Supplementary Material

Additional file 1 **Figure S1**. **S**imilarity of the query set with the NCBI database. Click here for file

Additional file 2 **Figure S2**. Distribution of the cut off for the e-value after blastx to NCBI nr. Click here for file

Additional file 3 **Figure S3**. Species distribution chart of kumquat transcripts after blastx to NCBI nr. Click here for file

Additional file 4 **Table S1**. Kumquat GO distribution analysis with sequence IDs, descriptions and annotation to each gene ontology category. Click here for file

Additional file 5 **Figure S4**. A maSigPro enriched Multilevel Pie chart illustrating the distribution of Molecular Functions within the statistically significant (P < 0.025 in single t-test) kumquat expressed genes on the chip. Click here for file

Additional file 6 **Figure S5**. A chart of the Cellular Components distribution within the maSigPro enriched statistically significant (P < 0.025 in single t-test) kumquat expressed genes in the chip. Click here for file

Additional file 7 **Table S2**. Differentially expressed ASCA-selected list of genes, at statistically significant levels, including p values, M values. Click here for file

Additional file 8 **Table S3**. Differentially expressed maSigPro-selected list of genes, including p values, M values. Click here for file

Additional file 9 **Table S4**. Combined analysis of statistically significant maSigPro+ASCA selected genes (P value≤ 0.05). Click here for file

Additional file 10 **Figure S6**. Quantitative realtime PCR (qRT-PCR) analyses of six selected kumquat ESTs (AAM60932 ABM67698, AA089566, AAk81874, AAC35981, AAV91900) in kumquat inoculated with *Xanthomonas axonopodis *pv. *citri *strain using a (5 × 10^8^cfu/ml) concentration of the Miami A strain X04-59. Leaf tissue was sampled for both inoculated and mock-inoculated plants at 0, 6, 24, 48, 72 and 120 hpi. (An average of three independent biological replications). Click here for file

Additional file 11 **Table S5**. Sequence data from this work have been deposited in the NCBI Genbank database libraries [Genbank: GW687757to GW690680]. Click here for file
