# Supplementary figures and images for: Fortunella margarita Transcriptional Reprogramming Triggered by Xanthomonas citri subsp. citri
Source: BMC Plant Biol. 2011 Nov 11;11:159. doi: 10.1186/1471-2229-11-159 (PMC3235979; doi:10.1186/1471-2229-11-159)

# E-value distribution

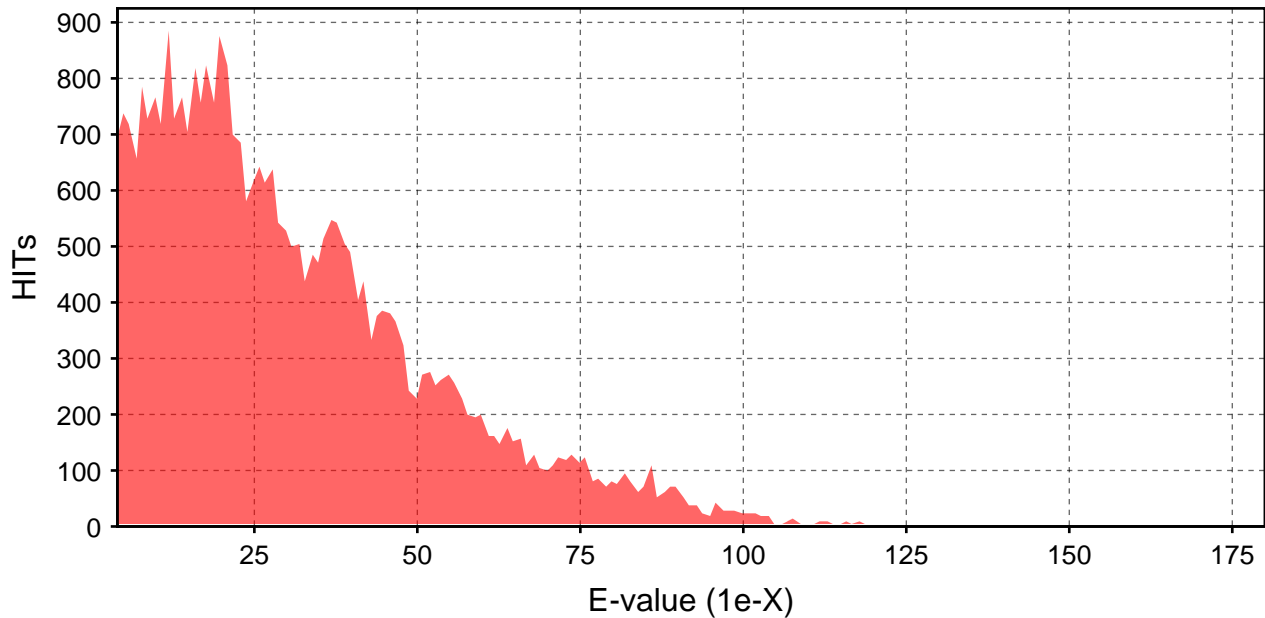

Supplement: Additional file 1 — Figure S1. Similarity of the query set with the NCBI database. [file 1471-2229-11-159-S1.PDF]

# Sequence similarity distribution

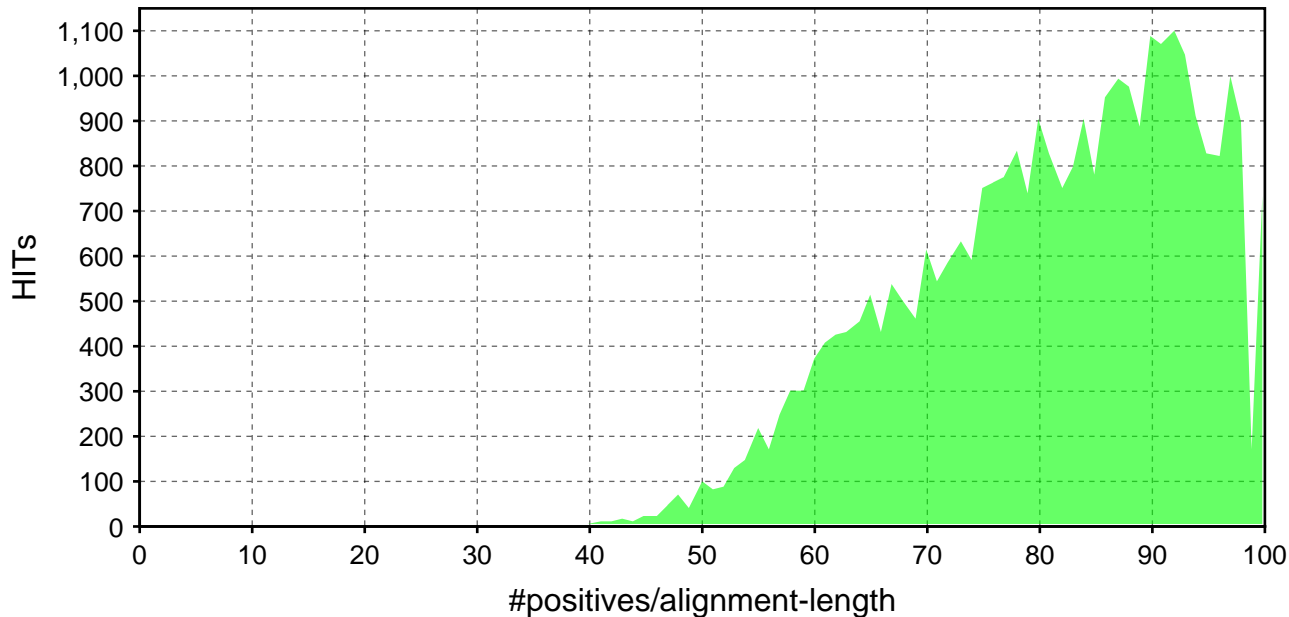

Supplement: Additional file 2 — Figure S2. Distribution of the cut off for the e-value after blastx to NCBI nr. [file 1471-2229-11-159-S2.PDF]

# Top-Hit species distribution

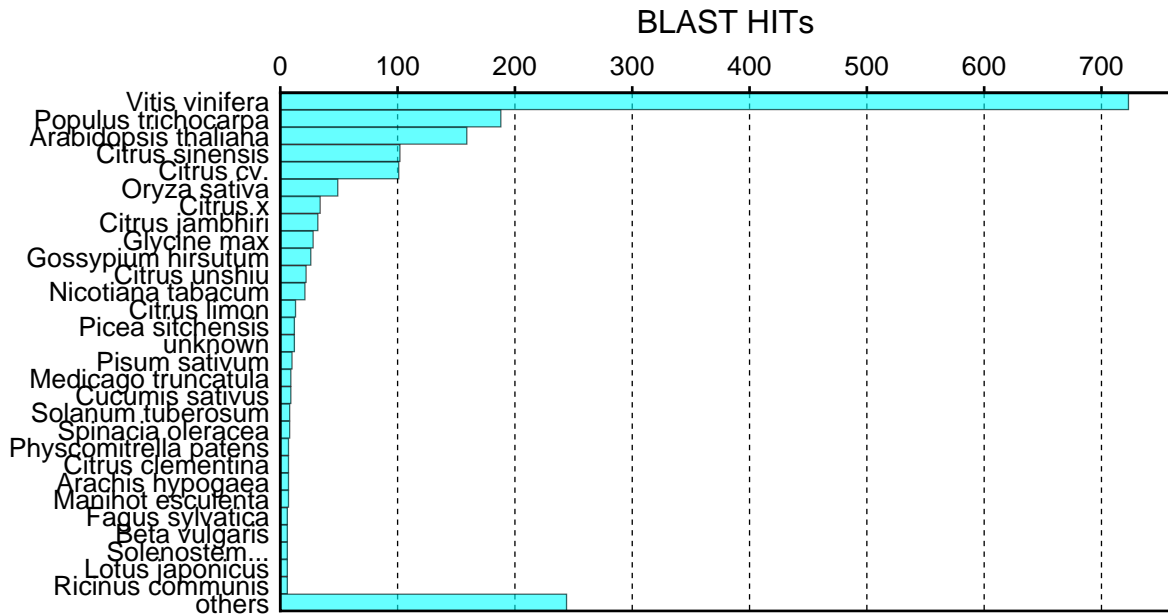

Supplement: Additional file 3 — Figure S3. Species distribution chart of kumquat transcripts after blastx to NCBI nr. [file 1471-2229-11-159-S3.PDF]

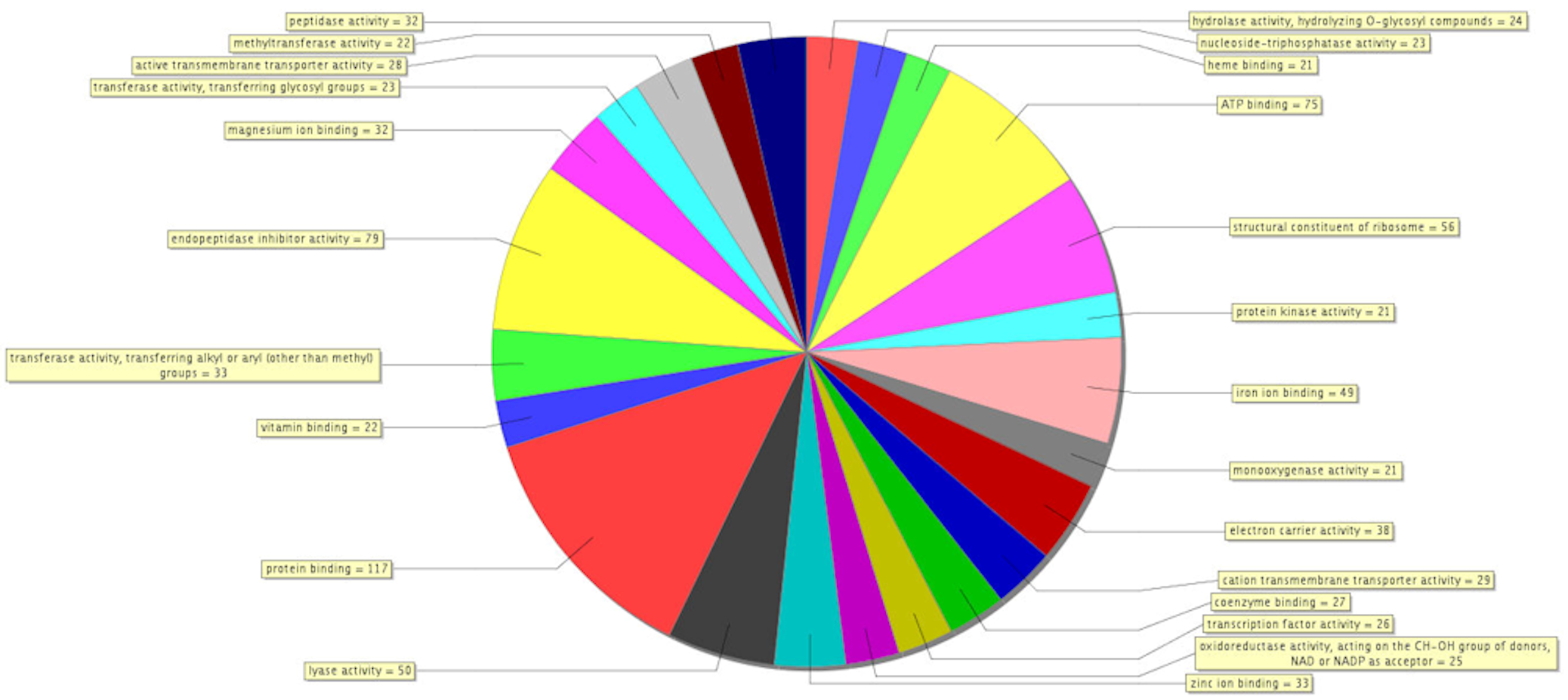

Supplement: Additional file 5 — Figure S4. A maSigPro enriched Multilevel Pie chart illustrating the distribution of Molecular Functions within the statistically significant (P < 0.025 in single t-test) kumquat expressed genes on the chip. [file 1471-2229-11-159-S5.TIFF]

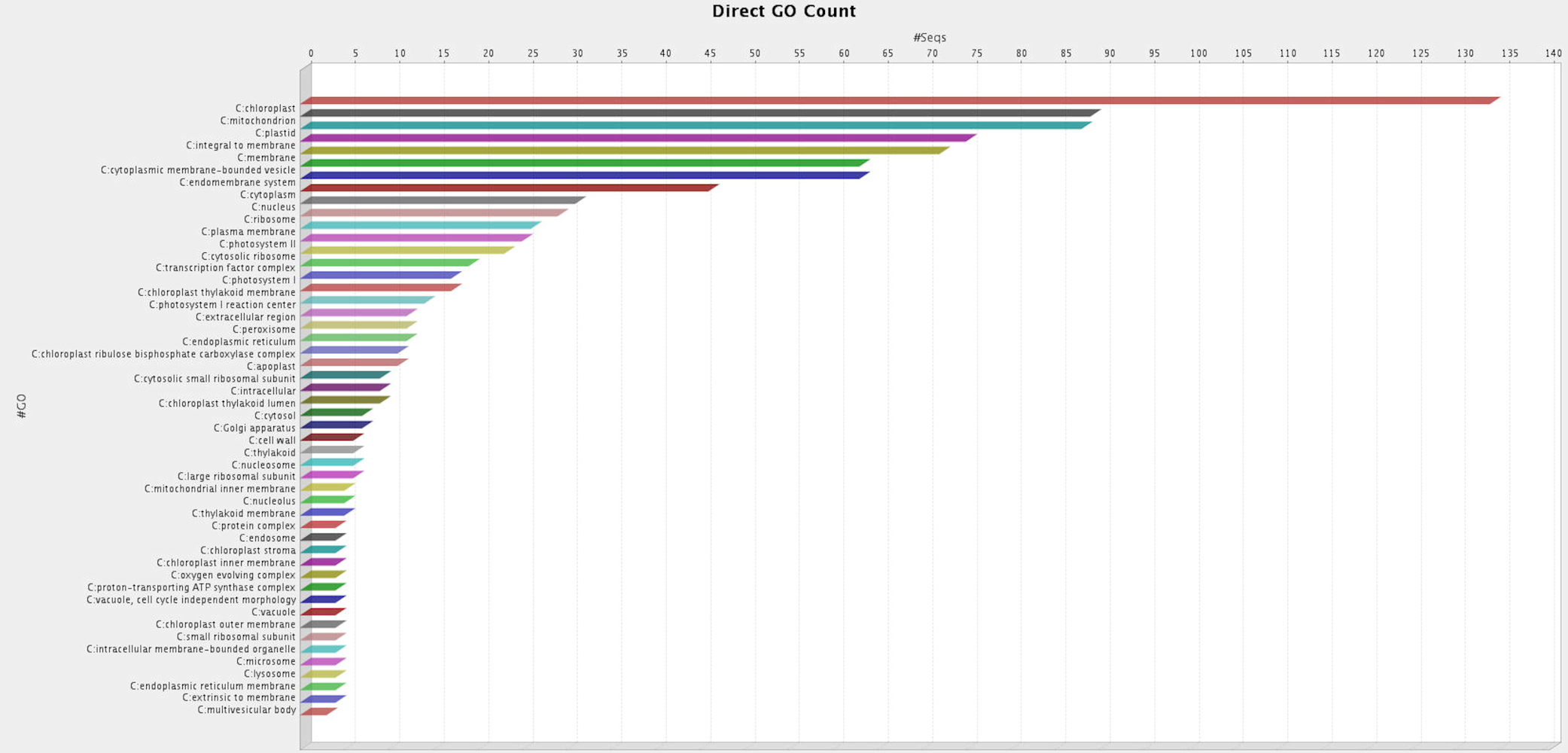

Supplement: Additional file 6 — Figure S5. A chart of the Cellular Components distribution within the maSigPro enriched statistically significant (P < 0.025 in single t-test) kumquat expressed genes in the chip. [file 1471-2229-11-159-S6.TIFF]

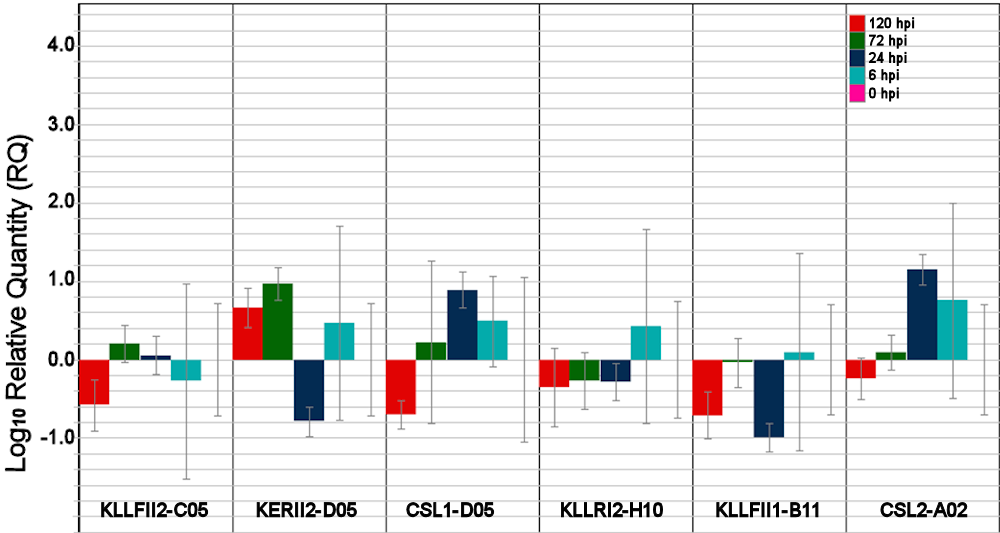

Supplement: Additional file 10 — Figure S6. Quantitative realtime PCR (qRT-PCR) analyses of six selected kumquat ESTs (AAM60932 ABM67698, AA089566, AAk81874, AAC35981, AAV91900) in kumquat inoculated with Xanthomonas axonopodis pv. citri strain using a (5 × 10^8^cfu/ml) concentration of the Miami A strain X04-59. Leaf tissue was sampled for both inoculated and mock-inoculated plants at 0, 6, 24, 48, 72 and 120 hpi. (An average of three independent biological replications). [file 1471-2229-11-159-S10.TIFF]
